# For patients with breast cancer, geographic and social disparities are independent determinants of access to specialized surgeons. A eleven-year population-based multilevel analysis

**DOI:** 10.1186/1471-2407-12-351

**Published:** 2012-08-13

**Authors:** Julie Gentil, Tienhan Sandrine Dabakuyo, Samiratou Ouedraogo, Marie-Laure Poillot, Olivier Dejardin, Patrick Arveux

**Affiliations:** 1 Côte d’Or Breast and Gynaecological Cancers Registry, Centre de Lutte Contre le Cancer Georges-François Leclerc, 1 rue Professeur Marion, 21000, Dijon, France; 2 EA 4184, Faculty of Medicine, University of Burgundy, 7 boulevard Jeanne d’Arc, 21000, Dijon, France; 3 Cancers & Preventions INSERM U1086 Faculté de médecine Université de Caen, Avenue de la Côte de Nacre, 14032, Caen, Cedex, France

## Abstract

**Background:**

It has been shown in several studies that survival in cancer patients who were operated on by a high-volume surgeon was better. Why then do all patients not benefit from treatment by these experienced surgeons? The aim of our work was to study the hypothesis that in breast cancer, geographical isolation and the socio-economic level have an impact on the likelihood of being treated by a specialized breast-cancer surgeon.

**Methods:**

All cases of primary invasive breast cancer diagnosed in the Côte d’Or from 1998 to 2008 were included. Individual clinical data and distance to the nearest reference care centre were collected. The Townsend Index of each residence area was calculated. A Log Rank test and a Cox model were used for survival analysis, and a multilevel logistic regression model was used to determine predictive factors of being treated or not by a specialized breast cancer surgeon.

**Results:**

Among our 3928 patients, the ten-year survival of the 2931 (74.6 %) patients operated on by a high-volume breast cancer surgeon was significantly better (LogRank p < 0.001), independently of age at diagnosis, the presence of at least one comorbidity, circumstances of diagnosis (screening or not) and TNM status (Cox HR = 0.81 [0.67-0.98]; p = 0.027). In multivariate logistic regression analysis, patients who lived 20 to 35 minutes, and more than 35 minutes away from the nearest reference care centre were less likely to be operated on by a specialized surgeon than were patients living less than 10 minutes away (OR = 0.56 [0.43; 0.73] and 0.38 [0.29; 0.50], respectively). This was also the case for patients living in rural areas compared with those living in urban areas (OR = 0.68 [0.53; 0.87]), and for patients living in the two most deprived areas (OR = 0.69 [0.48; 0.97] and 0.61 [0.44; 0.85] respectively) compared with those who lived in the most affluent area.

**Conclusions:**

A disadvantageous socio-economic environment, a rural lifestyle and living far from large specialized treatment centres were significant independent predictors of not gaining access to surgeons specialized in breast cancer. Not being treated by a specialist surgeon implies a less favourable outcome in terms of survival.

## Background

As shown in many studies, being treated in a specialized medical centre or by a high-volume surgeon is an independent prognostic factor for survival in patients with cancer, breast cancer particularly [1-9]. Moreover it is now well known that the socio-economic level is also an independent factor of survival in women with breast cancer [10-13]. For some cancers, socioeconomic deprivation and the distance to reference care centres have been studied and shown to be predictive factors of access to specialized care [14,15], but to our knowledge this has not been studied for breast cancer. In addition, there are disparities between a country’s socioeconomic level and rural/urban distribution: in the United States of America and the United Kingdom, city centres tend to be occupied by the most deprived people, whereas the wealthy live in suburbs around the cities. In France, it is the other way around: the most privileged classes live in the centre of big cities, whereas the most deprived people live further away in the suburbs or in rural towns. This study is one of the first to analyse the impact of surgeons’ experience on survival and the link between social characteristics and access to specialized surgeons.

In our population, we first studied the impact on survival of being operated on by a specialized breast cancer surgeon. We then determined whether the educational, social and economic environment of patients, the fact of living in a town or in the countryside, and the distance to the nearest reference care centre, independently of individual characteristics, such as age or stage at diagnosis, had an impact on being treated by one of the specialized breast cancer surgeons, all of whom work in one of the two reference care centres of the district.

## Methods

### Patients

We used the data from the Breast Cancer Registry of Côte d’Or, which has collected exhaustive and continuous medical information on all patients with breast cancer in the French administrative district of Côte d’Or, since 1982. The registry is part of the FRANCIM Network and as such, its exhaustiveness and data quality are regularly checked by the French Institute of Health and Medical Research (INSERM) and the International Agency for Research on Cancer (IARC). All cases of primary invasive breast cancer diagnosed over a period of eleven years, between 1st January 1998 and 31st December 2008 in women living in the Côte d’Or, were included. The Breast and Gynaecologic Cancer Registry of Cote d’Or was approved by the CNIL (National Commission on Informatics and Liberties) for the collection and recording of data for research purposes (authorization number DR-2012-038).

### Data collection and studied variables

High-volume surgeons were defined as those who had performed more than 100 breast cancer operations (from 130 to 602) during the study period: 8 (8.7 %) surgeons accounted for 74.6 % of all interventions. They all worked in one of the two reference care centres of the district, four in the private centre and four in the public centre, both located in the regional capital. Reference cancer care centres have been defined by the French Public Health authorities. In Côte d’Or it corresponds to the two highest-volume centres for breast cancer treatment, where more than 90 % of primary breast cancer surgery is performed.

#### Individual data

Age at diagnosis, year of diagnosis, TNM stage, the presence of at least one comorbid condition (diabetes, arterial hypertension, obesity, neurological and psychiatric diseases), time to travel to the nearest reference care centre by car and the circumstances of diagnosis were collected. All of the patients were staged according to the system described in the 5th TNM edition [16]. Staging was based on pathological findings; clinical information was used when pathological data were missing. The T stage was divided into three classes: T1, T2 and T3-T4, and the N and M stages into two classes, N+/N- and M+/M-. Time to the nearest cancer reference care centre was calculated using MAPINFO 9.1 (MapInfo Corporation), CHRONOMAP (Magellan engineering) and the Multinet Teleatlas road database, and divided into four classes: less than 10 minutes, 10 to 20 minutes, 20 to 35 minutes and more than 35 minutes. The circumstances of diagnosis were divided into 2 classes: patients who were diagnosed with cancer within the context of a screening program, and those with tumours discovered on clinical symptoms.

The year used for place of residence variables was the year of diagnosis.

#### Aggregate data

For all patients, we collected the INSEE (National Institute for Statistics and Economic Studies) socioeconomic aggregate data of their residence IRIS (Merged Islet for Statistical Information) and we calculated the Townsend index for each IRIS. The Townsend Index is a deprivation score calculated from four aggregated variables: the percentage of economically active residents aged 16-59/64 who are unemployed, the percentage of private households that do not possess a car, the percentage of private households that are not owner-occupied and the percentage of private households with more than one person per room [17] . The Townsend index was divided into quintiles. We also studied access to a reference surgeon according to whether the place of residence was in a rural or urban zone. A rural zone was defined by the Insee, as an IRIS including fewer than 10,000 inhabitants and not located in an urban area.

### Statistical analysis

Continuous variables were described as means, standard deviations and medians. Qualitative variables were given as percentages.

Survival was calculated from the date of diagnosis to the date of death or last follow-up. Overall survival was estimated using the Kaplan Meier method, and survival curves were compared using the Log-Rank test. For multivariate analysis, the Cox regression model was applied. Hazard Ratios (HR) and their 95 % confidence intervals were calculated. Age and stage at diagnosis, year of diagnosis, the presence of comorbidities and the circumstances of the diagnosis (screening or not) were included in the multivariate model as adjustment variables. Multivariate Cox proportional hazards’ modelling was applied to assess the independent prognostic effect for crude survival.

Our population presented a clear multilevel structure with patients (level 1) nested within the IRIS (level 2). The association between geographical and socioeconomic factors and being operated on by a high-volume surgeon was investigated using a multilevel logistic model. Univariate logistic regression was performed, giving p-values and Odds Ratios with their 95 % confidence intervals. All variables with a p value less than 0.05 were included in the multivariate model, always with age and stage as adjustment variables. Multivariate analysis of the predictive factors associated with being operated on by a high-volume breast-cancer surgeon was carried out using a multilevel logistic regression analysis: individual data were level one, aggregate data were level two. Level 2 variance and the variance partition coefficient, which represents the percentage of variance explained by the level 2, are given.

All analyses were carried out using SAS v 9.1 software and the final significance level was set at p < 0.05.

## Results

### Population

Our eleven-year registry population included 4646 patients. Among them, 3956 (85.1 %) had undergone surgery for their primary tumour. For 28 patients, the surgeon’s name was missing; these patients were therefore excluded. The characteristics of the 3928 remaining patients, according to the type of surgeon (high-volume or not) are presented in Table 1. The mean age of our patients was 60.1 years old, and 2931 (74.6 %) were operated on by a high-volume surgeon. The proportion of operations by year in the two groups was 69.3 % in 1998 and then 77.6, 75.3, 71.7, 72.6, 66.6, 73.0, 79.3, 79.5, 77.3 and 78.8 % for consecutive years between 1999 and 2008, respectively.

**Table 1:** Characteristics of patients according to the surgeon class (high-volume or not)

	**Total N = 3928**		**High-volume surgeon N = 2931**		**Others N = 997**		**P for heterogeneity**
	**N**	**%**	**N**	**%**	**N**	**%**	
**Individual data**							
**Age**							
< 50 years old	946	24.1	702	23.9	244	24.5	<0.001
50 to 74 years old	2438	62.1	1857	63.4	581	58.3	
> 74 years old	544	13.8	372	12.7	172	17.2	
**Circumstances of diagnosis**							
Screening	1907	51.1	1501	53.2	406	44.8	<0.001
Not screening	1823	48.9	1323	48.8	500	55.2	
Unknown	198						
**T Stage - size**							
T1	2875	75.7	2185	77.1	690	71.6	<0.001
T2	767	20.2	548	19.3	219	22.7	
T3 and T4	156	4.1	101	3.6	55	5.7	
Unknown	130						
**N Stage - nodes**							
N0	2591	68.2	1936	68.2	655	68.1	0.962
N1 and more	1211	31.8	904	31.8	307	31.9	
Unknown	126						
**M Stage - metastasis**							
M0	3809	98.2	2843	98.3	966	98.0	0.500
M1	69	1.8	49	1.7	20	2.0	
Unknown	50						
**At least one comorbidity**							
Yes	861	21.9	627	21.4	234	23.5	0.171
No	3067	78.1	2304	78.6	763	76.5	
**Time to the nearest reference cancer care centre**							
< 10 minutes	1153	29.3	917	31.3	236	23.7	<0.001
10 to 20 minutes	1049	26.7	831	28.4	218	21.8	
20 to 35 minutes	599	15.3	438	14.9	161	16.2	
> 35 minutes	1127	28.7	745	25.4	382	38.3	
**Aggregate data**							
**Place of residence**							
Rural	1067	27.2	746	25.5	321	32.2	<0.001
Urban	2860	72.8	2184	74.5	676	67.8	
Unknown	1						
**Townsend index**							
Quintile 1 (most affluent)	323	8.2	260	8.9	63	6.3	0.021
Quintile 2	557	14.2	432	14.8	125	12.5	
Quintile 3	484	12.3	363	12.4	121	12.1	
Quintile 4	722	18.4	531	18.1	191	19.2	
Quintile 5 (most deprived)	1839	46.9	1342	45.8	497	49.9	
Unknown	3						

### Follow-up and survival according to whether or not the patient was operated on by a high-volume surgeon

The follow-up was available for 3924 (99.9 %) patients: 3365 (85.8 %) women were still alive and 559 (14.2 %) had died.

**Figure 1:**
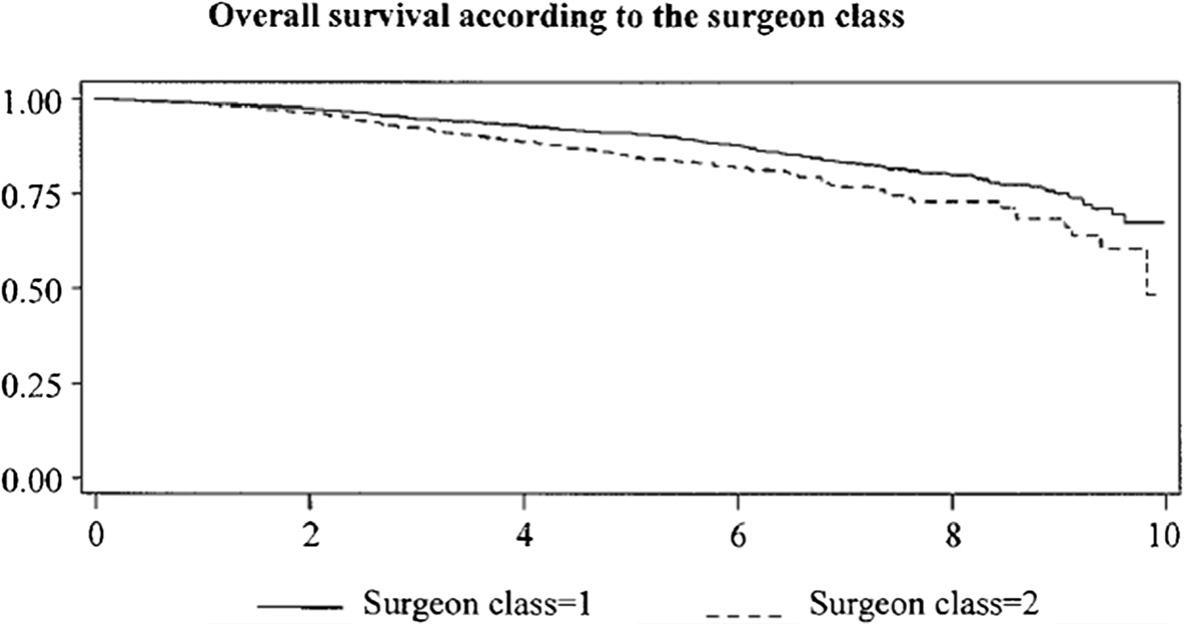
Overall survival curves (Kaplan-Meier) according to surgeon class, high-volume breast cancer surgeon (surgeon class = 1) or not (surgeon class = 2) (p < 0.001).

Figure 1 shows the overall survival curves (Kaplan-Meier) according to the surgeon class: high-volume breast-cancer surgeon (surgeon class = 1) or not (surgeon class = 2). Table 2 shows the proportions, the Log-Rank test and the results of the Cox multivariate analysis for overall survival according to the surgeon’s class.

**Table 2:** Univariate and multivariate analyses of overall survival according to the surgeon class (N = 3928)

	**Overall survival (%)**	**Univariate analysis Log-Rank p-value**	**Cox Multivariate analysis**	
			Hazard ratio (95% CI*)	**p-value**
**Surgeon class**				
High-volume	87.2	< 0.001	0.81 (0.67-0.98)	0.027
Others	81.6		1	
**Age at diagnosis**				
< 50 years old	90.0	< 0.001	0.87 (0.68-1.11)	0.267
50 to 74 years old	89.6		1	
> 74 years old	61.2		3.31 (2.71-4.04)	< 0.001
**T stage**				
T1	90.4	< 0.001	1	
T2	76.6		1.67 (1.35-2.05)	< 0.001
T3 or 4	51.9		2.70 (2.00-3.63)	< 0.001
**Node status**				
N0	90.4	< 0.001	1	< 0.001
N+	76.2		1.92 (1.58-2.32)	
**Metastasis status**				
M0	86.8	< 0.001	1	< 0.001
M+	34.8		3.32 (2.37-4.64)	
**At least one comorbidity**				
Yes	87.5	< 0.001	1.04 (0.85-1.26)	0.720
No	79.7		1	
**Circumstances of diagnosis**				
Screening	93.0	< 0.001	0.54 (0.43-0.68)	< 0.001
Clinical symptoms	83.1		1	

* CI: Confidence Interval.

The multivariate survival analysis highlighted the fact that patients who were operated on by a high-volume breast-cancer surgeon had significantly better survival (Hazard ratio HR = 0.81, 95%CI = [0.67-0.98]; p = 0.027), independently of age, year of diagnosis, TNM status, the presence of at least one comorbidity and the circumstances of diagnosis. As expected, age class and T, N and M status were also significant independent prognostic factors.

The presence of at least one comorbidity was a significant factor in univariate analysis, but no longer so in multivariate analysis. Indeed, it is strongly linked to the size of the most advanced tumour, to a positive N stage and the third age group (correlation coefficient p < 0.001 for all).

### Prognostic factors of being operated on by a high-volume breast-cancer surgeon

The univariate logistic regression model, which analyzed the relationship between the geographic and socioeconomic characteristics of patients and the likelihood of being operated on by a high-volume surgeon, showed that all variables were significant, except for node and metastasis status, which were then excluded from the multivariate analysis. The results of the univariate and multilevel logistic regression analyses are presented in Table 3.

**Table 3:** Remoteness and patients’ socio-economic characteristics: univariate and multilevel logistic regression analysis as predictive factors of being operated on by a high-volume breast cancer surgeon

	**Univariate logistic regression analysis**		**Multilevel logistic regression analysis**	
	Odds ratio (95 % CI*)	**p-value**	Odds ratio (95 % CI*)	**p-value**
**Individual data**				
**Age**				
< 50 years old	0.90 (0.76; 1.07)	<0.001		0.179
50 to 74 years old	1			
> 74 years old	0.68 (0.55; 0.83)			
**T Stage - size**				
T1	1	<0.001		0.189
T2	0.79 (0.66-0.95)			
T3 and T4	0.58 (0.41-0.81)			
**Circumstances of diagnosis**				
Screening	1	<0.001	1	0.005
Not screening	0.72 (0.62; 0.83)		0.78 (0.66-0.93)	
**Time to go to the nearest reference cancer care centre**				
< 10 minutes	1	<0.001	1	<0.001
10 to 20 minutes	0.98 (0.80; 1.21)		0.86 (0.68-1.07)	
20 to 35 minutes	0.70 (0.56; 0.88)		0.56 (0.43-0.73)	
> 35 minutes	0.50 (0.42; 0.61)		0.38 (0.29-0.50)	
**Aggregate data**				
**Place of residence**				
Rural	0.72 (0.62; 0.84)	<0.001	0.68 (0.53-0.87)	0.002
Urban	1		1	
**Townsend index**				
Quintile 1 (most affluent)	1	<0.001	1	0.013
Quintile 2	0.84 (0.60; 1.18)		0.84 (0.58-1.21)	
Quintile 3	0.73 (0.52; 1.03)		0.74 (0.52-1.08)	
Quintile 4	0.67 (0.49; 0.93)		0.69 (0.48-0.97)	
Quintile 5 (most deprived)	0.65 (0.49; 0.88)		0.61 (0.44-0.85)	

* CI: Confidence Interval.

The circumstances of the diagnosis remained significant in multivariate analysis: detection of the cancer during a mass screening programme, or during individual screening was a predictor of being operated on by a specialized surgeon. Patients who lived 20 to 35 minutes, and more than 35 minutes from the centres were less likely to be operated on by one of the high-volume breast-cancer surgeons than were patients who lived less than 10 minutes by car from one of the two reference care centres of the district. (OR = 0.56 [0.43; 0.73] and 0.38 [0.29; 0.50] respectively; p < 0.001). This was also the case for patients living in a rural area (OR = 0.68 [0.53; 0.87]; p = 0.002). Similarly, patients living in the two most deprived areas were less likely to be operated on by one of the high-volume breast-cancer surgeons than were patients living in the most affluent area (OR = 0.69 [0.48; 0.97]) and 0.61 [0.44; 0.85] respectively; p = 0.013).

The adjusted total variance between patients in the IRIS can be partitioned into variance between IRIS and variance between patients within the IRIS. Total variance can be broken down as follows: variance in the individual level was calculated at 249.7 (standard error SE = 3.5), variance in level 2 was calculated at 25.5 (6.1), and the variance partition coefficient was equal to 0.09.

## Discussion

Since the Calman and Hile report in 1995 [18] , the trend in European countries is to concentrate care supply in specialized cancer-care centres. In France, the Cancer Plan and the ministerial decree of March 29^th^ 2007, which established the minimal threshold for cancer operations illustrates this intention to concentrate cancer care. This policy decision was based on the hypothesis that patients are more likely to receive high-quality treatment if their surgeon has experience in operating on their particular cancer. This hypothesis is confirmed in our study: survival in patients operated on by one of the highest-volume breast cancer surgeons was significantly higher than in others.

Our study has several limits. First of all, the survival analysis did not take into account all of the possible confounding factors. Moreover, in order to simplify the analysis, we established a threshold to define two classes of surgeons: we used a threshold of 100 operations, as we had done several survival analyses using various categories of reference surgeons: there was no significant difference among surgeons who had operated on more than 100 breast cancers, but there was a significant difference between the above surgeons and those who had done fewer than 100 operations, among whom there was no significant difference either.

In addition, in practice, among the 92 surgeons who had operated on at least one breast cancer between 1998 and 2008, the eight who had performed the most operations, in a regular manner during their years of practice, and were known to be surgeons of the department specialized in breast cancer surgery, were those who had done more than 100 operations each, the ninth and the following surgeons had all done less than 100 operations over the study period.

Nonetheless, we adjusted our multivariate survival analysis for age, year of diagnosis, TNM stage, comorbidities and the circumstances of the diagnosis, in order to minimize the number of confounding factors. In contrast, our analysis was not adjusted for other cancer treatments, notably chemotherapy and radiotherapy, which also have an impact on survival. But, very often, surgery is the first step in the treatment, and the surgeon or the centre the patient is referred to is the gateway to the rest of the treatment. Access to one of these surgeons is therefore a prognostic factor in breast cancer. More than a quarter of our patients was not referred to and did not see a specialized surgeon.

The results of our study suggest that remoteness from a reference care centre, meaning remoteness from a regional capital, and a socioeconomically deprived environment have an impact on access to surgeons specialized in breast cancer. The most deprived patients and patients who lived far from their regional reference care centre for breast cancer were less likely to be operated on by a high-volume surgeon. Our study showed that the probability of being operated on by a specialized surgeon is inversely proportional to the socio-economic level of the place of residence as determined by the IRIS. Though the difference was not statistically significant for the 2nd and 3rd quintiles in comparison with patients of the most affluent Townsend Index quintile, patients of the two most deprived quintiles were respectively 1.5 and 1.6 times more likely to be operated on by a non-specialist surgeon.

The same was true for remoteness; there was no significant difference between patients who lived less than 10 minutes by car, and those who lived between 10 and 20 minutes by car from one of the two reference treatment centres of the Cote d’Or. However, patients who lived 20 to 35 and more than 35 minutes away from a reference care centre were respectively 1.8 and 2.6 times more likely to be operated on by a non-specialist surgeon than were patients living closest to the centres. In our study, the likelihood of being treated by a high-volume surgeon decreased with distance from the specialized centre. This means that unfavourable geographical and social characteristics have to be considered as potential predictive factors of a less than optimal surgical result, of potential recurrence and worse survival, as suggested by a French report on the risk of being less well treated in a low-volume hospital [19]. Moreover, patients who are less likely to be treated in reference centres are generally the same as those who are less likely to receive optimal overall disease management. Geographical and socioeconomic disparities remained significant even after adjustment for TNM status and age at diagnosis. Our findings showed a trend towards the results of similar studies about cancer treatment in reference centres, especially for colorectal cancer [14,15].

Another interesting point is that women who were diagnosed within the context of a screening program were significantly more likely to be operated on by a high-volume surgeon than were patients who were diagnosed on clinical symptoms. This parameter was included in the analysis as an adjustment variable to study the hypothesis that patients with disadvantageous geographic and socio-economic characteristics were less likely to be referred to a high-volume surgeon because they were less likely to take part in mass screening programs. The result underpins the hypothesis that women who take part in mass screening programs for breast cancer are indeed more likely to be operated on by a specialized surgeon and this independently of remoteness and socio-economic level of their place of residence.

This raises other hypotheses on cancer detection and treatment patterns that need to be explored in specific studies.

## Conclusions

Though we showed that socioeconomic deprivation and remoteness were independent predictors of access to a specialized surgeon, and as such, could be linked to survival, we were unable to explain why underprivileged patients or those living far from reference care centres were less likely to be referred to specialized surgeons. Though it can be understood that patients who live far from a reference care centre may prefer to be treated locally rather than travel, it is difficult to explain why the most deprived patients, independently of the distance, were less likely to receive specialized care.

Nevertheless, social and geographical disparities with regard to access to care for patients with breast cancer remain, and these disparities can affect the chances of survival. Improvements need to be made in the quality of care in non-reference care centres, or in the distribution of specialized care centres around the country, to avoid aggravating these differences.

## Competing interests

The authors declare that they have no competing interests.

## Authors’ contributions

JG and PA conceived and designed the study; JG, TSD, SO and MLP participated in the quality control and data acquisition; JG and OD performed statistical analyses and interpreted the data; JG and TSD wrote the manuscript. All the authors read and approved the final manuscript.

## Pre-publication history

The pre-publication history for this paper can be accessed here:

http://www.biomedcentral.com/1471-2407/12/351/prepub
